# Can cash transfers protect mental health? Evidence from an observational cohort of children and adolescents living in adverse contexts in Brazil

**DOI:** 10.1192/j.eurpsy.2025.10109

**Published:** 2025-09-24

**Authors:** Cristiane Silvestre Paula, Carolina Ziebold, Isabel A.S. Bordin, Alicia Matijasevich, Sara Evans-Lacko

**Affiliations:** 1 Programa de Pos-Graduação em Ciências do Desenvolvimento Humano, Human Developmental Sciences Program, Universidade Presbiteriana Mackenzie, São Paulo, Brazil; 2 Department of Psychiatry, Federal University of Sao Paulo: Universidade Federal de Sao Paulo, São Paulo, SP, Brazil; 3 Departamento de Medicina Preventiva, Faculdade de Medicina FMUSP, Universidade de Sao PauloUniversidade de Sao Paulo, São Paulo, SP, Brazil; 4 Care Policy and Evaluation Centre, Department of Health Policy, London School of Economics and Political Science, London, UK

**Keywords:** adverse childhood experiences, behavior problems, children and adolescents, mental health, monetary incentive, protective factors, vulnerable populations

## Abstract

**Background:**

Youth exposed to poverty and adversities like violence are at higher risk of mental health problems (MHP), but whether antipoverty interventions can reduce this risk remains unclear. We examined the association between participation in the Brazilian Cash Transfer Program (BFP) and mental health of children/adolescents exposed to different levels of adversity.

**Methods:**

Observational study using nearest-neighbor propensity score matching to compare BFP participants and non-participants from the Itaboraí study, a community-based cohort of 1,189 children/adolescents (6–15 years) assessed at two waves (meaninterval: 12.9 months).Measures included the Child Behaviour Checklist (CBCL) externalizing, internalizing, and total problems scales; an adversity score derived from a confirmatory factor analysis on violence victimization at home (WorldSAFE), school (threat/maltreatment/being chased by peers) and community (Survey of Exposure to Community Violence), and stressful life events (UCLA Posttraumatic Stress Disorder Reaction Index); and BFP exposure for at least 12 months (yes/no). Latent change score models tested whether BFP participation predicted changes in CBCL T-scores, moderated by adversity levels.

**Results:**

A total of 330 BFP participants were matched with 330 non-participants with similar sociodemographic characteristics. Decreases in total (b=−0.124, SE=0.034, p<0.001), externalizing (b=−0.122, SE=0.036, p=0.001), and internalizing problems (b=−0.141, SE=0.033, p<0.001) between baseline and follow-up were observed among BFP participants exposed to higher levels of adversity compared with non-participants.

**Conclusions:**

BFP participation was associated with reduced MHP only among children/adolescents facing high adversity, suggesting the program may help break the cycle between poverty and mental health problems—but benefits are concentrated among the most vulnerable.

## Introduction

Mental health conditions are the leading cause of years lived with disability among young individuals [1, 2].

Multiple studies suggest that poverty and socioeconomic disadvantage are a consistent driver of mental health problems [3, 4]. There are some well-established theoretical frameworks such as Social Causation Theory where poverty would lead to mental health problems, while growing evidence suggests a cyclical relationship between poverty and mental health [5], where each factor influences the other [6–8].

Socioeconomic disparities, particularly pronounced during childhood/adolescence [9], underscore the need for early intervention to mitigate mental health consequences, especially among those in vulnerable situations [10]. Yet, despite available evidence-based interventions for youth mental health [11, 12], few individuals have access [13], particularly in low- and middle-income countries (LMICs) such as Brazil [14–16].

Cash transfer programs (CTPs) have emerged as an effective poverty alleviation strategy [17]. These programs are widely implemented [18, 19] and have the potential to improve mental health [20–22]. Numerous studies have shown that CTPs, beyond alleviating poverty, also increase healthcare utilization, improve child nutrition, and boost school attendance around the globe [18, 23, 24], particularly in Brazil [25–27]. Recent research suggests modest support for mental health benefits of CTPs [28, 29]. However, the findings are mixed, with several gaps and inconsistencies in the literature. These discrepancies likely arise because CTPs do not necessary directly address the mechanisms through which poverty undermines mental health [6, 30, 31]. Evidence on children/adolescents is in the same direction but more limited than for adults, with most studies focusing on internalizing symptoms or wellbeing and fewer examining externalizing symptoms or comparing types of problems [31–33].

Given the mixed evidence regarding the impacts of CTPs on child and adolescent mental health, we hypothesize that these programs may not benefit all individuals equally. Drawing on theories from behavioral economics and cognitive psychology, and considering the social determinants of mental health, Evans-Lacko et al. [6] propose that CTPs may influence key mediating pathways at the family and community levels that positively affect youth mental health. In this context, we suggest that children and adolescents exposed to higher levels of adversity – particularly at the family/household level (e.g., domestic violence, caregiver stress) and community level (e.g., violent crime, unsafe school environments) – may derive greater benefit from such programs. For example, when transfers are directed to female caregivers, CTPs may help reduce family stress, conflict, and domestic violence [34, 35]. At the community level, they may promote social cohesion, reduce violence, and lessen income inequality [36–38]. Such improvements in the family and social context may, in turn, enhance mental health outcomes for the most vulnerable youth.

Hence, we investigate whether participation in the Brazilian Bolsa Família CTP (BFP) is associated with reductions in mental health problems – overall, and specifically in externalizing and internalizing problems – and whether these associations vary by exposure to higher levels of adversity, including violence in the community, school, and home, as well as other stressful life events. We hypothesize that individuals facing multiple adversities may experience greater mental health benefits associated with BFP participation. This observational approach allows us to explore differential associations of CTPs across subgroups, offering new insights into their potential protective role.

## Methods

We used data from a subsample of the Itaboraí Youth Study, a longitudinal cohort comprising a probabilistic community-based sample of children/adolescents aged 6–15 years at baseline (n = 1,409) and 7–17 years at follow-up (n = 1,189) from low-income backgrounds [39]. Briefly, the study used a three-stage sampling procedure: random sampling of census units, random sampling of eligible households, and random selection of a target child. Trained interviewers collected information on sociodemographics, violence exposure, and mental health through face-to-face individual interviews with biological/adoptive/stepmothers in households at baseline and follow-up in 2015 (mean interval of 12.9 months and an 84.4% retention rate). The study received approval from Brazil’s National Committee for Ethics in Research Number 25000.182992/2011–76. All mothers provided written informed consent.

### Study site

Itaboraí is a low-income medium-size city in Rio de Janeiro State, Brazil, with an estimated population of 229,007 in 2015, of which 98% residing in urban areas and nearly one-third under 20. Over one-third of Itaboraí’s population lives in poverty, with 51.3% of residents over 24 not completing 8 years of schooling compared to 42% nationwide [40]. The city has high homicide rates among 1–19 year olds (27.2 per 100,000 in 2010), compared to the state (17.2/100,000) and national rates (13.8/100,000) [41]. In 2015, 65.8% of the population received benefits from the BFP, significantly higher than the national average (22%) [42].

### Measures

#### Exposures

##### Adversity at baseline

Exposure to adversity was measured using standardized factor scores (continuous variable) from a confirmatory factor analysis (CFA) on a single latent measure comprising items from the following questionnaires:

- Brazilian version of the World Studies of Abuse in the Family Environment Core Questionnaire [43] which explores exposure to eight forms of severe physical punishment at home by one or both parents in the previous year.
- 11-item questionnaire selected from the Survey of Exposure to Community Violence that investigates exposure to community violence: beatings and muggings, forced entry, being chased, arrests, threats, knife attacks, shootings, and sexual molestation [44].
- Mother-perception of bullying through the question: “Was the child threatened, maltreated, or chased by peers at school in the past 12 months?” developed by the authors [39].
- Mothers reported on child’s exposure to stressful life events in the past 12 months: 15 items that are part of the evaluation of Post-Traumatic Stress Disorder (PTSD) according to DSM-IV criteria, validated in Brazil [45].

We conducted a CFA using Mplus version 8.6 [46] to examine whether items assessing exposure to stressful life events and violence – across home, community, and school contexts – could be represented by a single underlying latent construct, referred to as the “adversity factor.” Given the categorical nature of the items, we employed the weighted least squares means and variance adjusted estimator.

A one-factor solution was tested based on both theoretical rationale and parsimony. Theoretically, prior research suggests substantial overlap between different forms of adversity among children living in vulnerable contexts, supporting the plausibility of a unidimensional construct [47]. From a methodological standpoint, a one-factor model provides a more parsimonious explanation of the data, assuming adequate model fit [48]. Model fit was evaluated using established criteria: the root-mean-square error of approximation (RMSEA < 0.06), comparative fit index (CFI > 0.90), and Tucker–Lewis index (TLI > 0.90) [48].

Table 1 presents the standardized factor loadings of the adversity factor for each item. These loadings indicate the strength and direction of the relationship between each item and the underlying adversity factor, with higher loadings reflecting stronger associations. The one-factor model encompassing responses to all these questionnaires had good fit to the data (RMSEA = 0.035, CFI =0.932, TLI =0.928). These findings support the validity of modeling the selected adversity indicators as manifestations of a single latent factor. The CFA diagram is presented in Appendix 1.

**Table 1.** Confirmatory factor analysis: Standardized factor loadings of the adversity factor on each item

Item	Factor loading	SE	p
Building collapse	0.609	0.044	<0.001
Fire, flood, or other natural disaster	0.580	0.042	<0.001
Life-threatening accident	0.552	0.038	<0.001
Running over with serious injury	0.885	0.008	<0.001
Biking accident with serious injury	0.612	0.044	<0.001
Motorcycle accident with serious injury	0.689	0.038	<0.001
Car accident with serious injury	0.672	0.040	<0.001
Other traffic accident with serious injury	0.867	0.010	<0.001
Child saw a dead person victim of violence	0.458	0.035	<0.001
Child saw a dead person victim of accident	0.448	0.028	<0.001
Child received bad news about violent death or serious injury of a loved one	0.304	0.024	<0.001
Life-threatening illness of a close family member	0.206	0.021	<0.001
Death of a close family member	0.180	0.021	<0.001
Problems with alcohol or drugs of a close family member	0.277	0.022	<0.001
Close family member being arrested or having problems with the police	0.365	0.024	<0.001
Threatened, maltreated, or chased by peers at school	0.288	0.026	<0.001
Being mugged	0.566	0.040	<0.001
Someone has broken into or tried to force their way into the house or apartment	0.598	0.042	<0.001
Being chased by gangs or individuals	0.642	0.041	<0.001
Being picked-up/arrested by the police or taken to the police station	0.966	0.021	<0.001
Being threatened by someone with serious physical harm	0.586	0.041	<0.001
Suffering death threats	0.869	0.009	<0.001
Being beaten-up (outside home/school)	0.620	0.041	<0.001
Being around a shoot-out	0.459	0.033	<0.001
Mother hit him/her with an object such as a stick, bloom, cane, or belt	0.425	0.018	<0.001
Mother kicked him/her	0.688	0.035	<0.001
Mother choked him/her by putting hands (or something else) around his/her neck	0.816	0.013	<0.001
Mother smothered him/her with hand of pillow	0.879	0.007	<0.001
Mother burned, scalded, or branded him/her	0.979	0.015	<0.001
Mother beat him/her	0.551	0.025	<0.001
Mother beat him/her (hit over and over again with object or fist)	0.888	0.007	<0.001
Mother threatened him/her with a knife or gun	0.874	0.007	<0.001
Mother harmed him/her with a knife or gun	0.979	0.015	<0.001
Father hit him/her with an object such as a stick, bloom, cane, or belt	0.570	0.034	<0.001
Father kicked him/her	0.792	0.026	<0.001
Father choked him/her by putting hands (or something else) around his/her neck	0.893	0.008	<0.001
Father beat him/her	0.665	0.040	<0.001

*Note*: SE, standard error. Original items not included due to lack of participants with affirmative responses: being attacked or stabbed with a knife; being shot; being sexually molested by someone much older; father smothered him/her with hand of pillow; father burned, scalded, or branded him/her; father beat him/her (hit over and over again with object or fist); father threatened him/her with a knife or gun; and father harmed him/her with a knife or gun.

#### Bolsa Família participation at baseline

Eligibility for the BFP is based on per capita household income and household composition, including pregnant or breastfeeding mothers and children aged 0–17. Monthly payments, averaging US$ 59 per month in 2015, are made to mothers [49]. For the current study, mothers were asked: *Does your family receive BFP?* (yes/no) and, if yes, length of participation in the program (months). We classified as “exposed” those receiving the benefit for at least 12 months at follow-up ensuring a plausible impact changes in the child’s mental health.

#### Outcome: Changes in CBCL T-scores between baseline and follow-up

Child/adolescent emotional/behavioral problems were assessed using the Brazilian version of the Child Behavior Checklist (CBCL) with 118 items on a three-point Likert scale (0–3) [50]. We used US normative data to generate T-scores (mean 50, SD = 10). The questionnaire, completed by mothers comprises three scales: 1) internalizing behavior problems including anxious depressed, withdrawn depressed, and somatic complaints, 2) externalizing behavior problems including rule-breaking behavior and aggressive behavior, and 3) total behavior problems for the total score. The main outcome for the present study was the changes in total T-scores between baseline and follow-up. Negative values indicate a reduction in problems since baseline. Secondary outcomes included changes in externalizing and internalizing T-scores between baseline and follow-up.

#### Covariates

To mitigate potential confounding in comparing BFP participants and non-participants, we assessed the following key sociodemographic characteristics for matching:

- Maternal characteristics: age (years) and marital status (single or married/living with partner);
- Household characteristics: number of people residing in the household and number of children aged 6–15 years (eligible for participation in study). Socioeconomic status at wave two was assessed using the Associação Brasileira de Empresas de Pesquisa/Brazilian Association of Research Companies (ABEP) questionnaire, a widely used tool in Brazil. It considers factors like the number of home appliances and the educational level of the household head, generating a score from 0 to 100. According to the 2015 Brazilian criteria, scores ≤16 categorized households within the poorest stratum [51].
- Child characteristics: age (in years) and sex (male/female).

### Data analysis

#### Propensity score matching

Given the observational nature of the study and non-randomized nature of the BFP, establishing a valid comparison group of non-beneficiaries to estimate BFP is challenging [52]. To address this, we undertook a two-step process:

1. We excluded 69 participants categorized as high socioeconomic group based on ABEP criteria (scores > 45).
2. We used propensity score matching (PSM) to create comparable groups of BFP Beneficiaries and non-beneficiaries based on observed characteristics. Propensity scores were estimated using *probit* regression models incorporating factors significantly associated with program participation (p < 0.05) [53–55]. Each BFP participant was matched with a non-participant with the closest propensity score, using 1:1 nearest-neighbor matching (NNM) without replacement and a caliper (maximum distance between the PS of matched pairs) of 0.25 [53–55]. NNM is particularly suitable for longitudinal studies [46, 56]. To evaluate matching performance we: a) tested the balance of sociodemographic characteristics between beneficiaries and non-beneficiaries in the matched sample, b) measured the percentage of bias reduction post-matching, and c) reviewed density-distribution plots of propensity scores before and after matching, demonstrating matched participants had similar probabilities of BFP participation based on the sociodemographic profile [53].

#### Statistical analyses

After selecting the matched sample, we used normality plots to inspect the distribution of CBCL T-scores and standardized the adversity factor scores. Linear regression models were initially used to explore the association between 1) standardized adversity factor scores and total, internalizing, and externalizing CBCL T-scores at baseline and 2) BFP participation and standardized adversity factor scores at baseline.

Our main analysis focused on whether BFP participation at baseline moderated the association between adversity and increased CBCL T-scores over time. We used latent change score models (LCSMs) because they allow for: (1) robust identification of the average change between assessments while controlling for the initial outcome level and covariates, (2) consideration of individual variability in change over time, (3) adjustment of initial outcome levels based on BFP participation status and adversity scores, and (4) identification of predictors of change in the outcome variable [57, 58]. LCSMs are particularly effective for examining the interrelationship of different aspects involved in neurodevelopment [57]. The predictor of change tested across the three models – for total, internalizing, and externalizing CBCL T-scores – was the interaction between BFP participation at baseline and adversity factor scores. The model structure is illustrated in Appendix 2. For each model, a latent difference factor was defined to represent change in the outcome from baseline (T1) to follow-up (T2). This factor was specified with a fixed loading of 1 on the outcome at T2, indicating that it captures the difference component. An autoregressive path from the outcome at T1 to T2 was also fixed at 1, such that T2 was modeled as a function of the T1 score plus the latent change factor. This approach ensures that the latent change reflects deviation from baseline rather than serving as an independent predictor of T2 [57, 58].

To focus estimation on change rather than fixed or static levels, intercepts for the observed outcomes at T1 and T2 were fixed at zero. In turn, the mean of the latent change factor was freely estimated, providing an estimate of average within-person change over time. The residual variance of the outcome at T2 was fixed at zero, under the assumption that all variabilities in T2 scores are explained by T1 and the latent change factor. To model proportional change, a covariance was estimated between the latent change factor and the baseline score (T1), allowing for the possibility that change varied systematically based on initial levels [57, 58].

Baseline predictors – participation in the BFP and standardized adversity factor scores – were included as covariates predicting T1 scores. Additionally, the latent change factor was regressed on an interaction term between adversity and BFP participation to assess whether BFP moderated the association between standardized adversity factor scores and within-person change.

We report standardized estimates from the latent change score model. These coefficients represent the expected change in the outcome (in standard deviation units) associated with a one standard deviation change in the independent variable.

We used the maximum likelihood estimator. Good model fit in the LCSMs, was assessed using: CFI and TLI values ≥0.90 and RMSEA values <0.08 [48].

All analyses were performed using STATA version 17 [55], except for CFA and LCSMs which were conducted using Mplus version 8.6. [46]. A significance level a = 0.05, two tailed, was considered for main analyses.

#### Addressing potential bias due to attrition

Inverse probability weights (IPWs) were applied to mitigate attrition bias and ensure that the analytical sample remained representative of the original baseline sample. To estimate these weights, probit regression models were used to identify predictors of attrition based on baseline variables. As shown in Table 2, only maternal employment status was significantly associated with attrition. However, to minimize potential residual confounding, all relevant baseline variables were included in the prediction of attrition probabilities, which were then used to calculate propensity scores. IPWs were generated by assigning greater weight to participants with a lower probability of remaining in the study (i.e., complete cases were weighted inversely to their probability of being retained). These weights were incorporated as sampling weights in the specification of the LCSM, following the approach outlined by Asparouhov [59].

**Table 2.** Predictors of attrition at follow-up

	Participated at follow-up	Attrition	p value*
Factor, n (%)	1189 (84.4)	220 (15.6)	
Child’s sex			0.46
Male, n (%)	621 (52.2)	109 (49.5)	
Female	568 (47.8)	111 (50.5)	
Child’s age, mean (SD)	10.48 (2.87)	10.72 (2.83)	0.25
Mother/informant: age, mean (SD)	36.72 (8.25)	36.16 (7.77)	0.35
Number of children in the household aged 6–15 years, mean (SD)	1.38 (0.65)	1.41 (0.69)	0.55
Mother: worked for pay in last 30 days, n (%)			0.023
No	564 (47.4)	86 (39.1)	
Yes	625 (52.6)	134 (60.9)	
Mother living with a husband/partner, n (%)			0.35
No	357 (30.0)	73 (33.2)	
Yes	832 (70.0)	147 (66.8)	
Total CBCL T-scores, mean (SD)	50.00 (11.37)	48.75 (11.31)	0.13

* Probit regression models were used to identify predictors of attrition based on baseline study variables.

#### Sensitivity analyses

Sensitivity analyses were conducted to assess the robustness of the findings. First, we operationalized duration of BFP participation as a continuous variable (with non-beneficiaries assigned a duration of 0) instead of using a categorical variable. Second, we repeated the analyses using an alternative exposure definition, considering children as beneficiaries if they had received BFP support for at least 6 months. Third, analyses were performed using the full pre-matched sample to assess whether results were consistent prior to matching. Fourth, we conducted the main analyses on a matched sample that excluded ABEP socioeconomic scores and access to piped water in the propensity score matching, as these were collected during follow-up and could be influenced by post-intervention factors. Finally, we conducted the analyses removing the IPWs intended to handle attrition.

## Results

### Sample differences before and after PSM

Our analysis included 1,020 participants after excluding 69 individuals from higher socioeconomic groups, of which 373 (36.6%) were BFP beneficiaries. Table 3 presents sample characteristics and differences before PSM. Before matching, BFP beneficiaries were more likely to have mothers who were younger, unemployed, have more children, less access to piped water and lower purchasing power scores than non-beneficiaries. We found no differences between groups according to child’s sex, age, or mother’s marital status. In the unmatched overall sample, the mean total CBCL-T score was 48.5 (SD = 10.01) among non-beneficiaries and 52.39 (SD = 10.49) among beneficiaries. An unadjusted regression model indicated significantly higher scores among BFP participants (β = 3.85; 95% CI: 2.59–5.12; p < 0.001).

**Table 3.** Identification of factors related to BFP participation (n = 1,020 – Itaboraí Youth Study excluded high socioeconomic group)

	Non-BFP	BFP	p value
Factor, n (%)	747 (63.4)	373 (36.6)	
Child’s sex, n (%)			0.848
Male	390 (52.2)	197 (52.8)	
Female	357 (47.8)	176 (47.2)	
Mother: age, mean (SD)	38.05 (8.71)	36.71 (7.51)	**0.012**
Number of people residing in the household, mean (SD)	3.97 (1.10)	4.38 (1.28)	**<0.001**
Family purchase power score, mean (SD)	16.33 (5.77)	12.96 (4.68)	**<0.001**
Household receive water from the public system, n (%)			**0.045**
No	522 (69.9)	282 (75.6)	
Yes	225 (30.1)	91 (24.4)	
Mother: worked for pay in last 30 days, n (%)			**<0.001**
No	270 (36.1)	186 (49.9)	
Yes	477 (63.9)	187 (50.1)	
Mother living with a husband/partner, n (%)			0.266
No	267 (35.7)	146 (39.1)	
Yes	480 (64.3)	227 (60.9)	
Index child: age, mean (SD)	11.57 (2.86)	11.55 (2.85)	0.879
Number of children in the household aged 6–15 years, mean (SD)	1.31 (0.61)	1.54 (0.72)	**<0.001**

*Note*: BFP, Bolsa Família Program.

Following PSM, we achieved a balanced, matched sample of 660 participants, 330 BFP beneficiaries. This process addressed initial imbalances in sociodemographic characteristics observed between groups (Table 4). Quality checks, presented in Appendix 3, include (before and after matching): standardized percentage of bias across covariates (Figure 3.1 in Appendix 3.1), bias diagnosis before and after matching (Table 3.1 in Appendix 3.1), density plot of BFP participation propensity score (Figure 3.2 in Appendix 3.2), and density plot of family purchase power score (ABEP) (Figures 3.3 and 3.4 in Appendixes 3.3 and 3.4). These analyses demonstrate the robustness of PSM in removing differences in the distribution of covariates, increasing our confidence that the observed differences in outcomes could be attributed to BFP participation.

**Table 4.** Sociodemographic characteristics of the matched sample included in the study by BFP participation status (Itaboraí youth study n = 660)

	Total	Non-BFP	BFP	p value*
Factor, n (%)	660 (100)	330 (50)	330 (50)	
Child’s sex, n (%)				0.102
Male	335 (50.8)	157 (47.6)	178 (53.9)	
Female	325 (49.2)	173 (52.4)	152 (46.1)	
Child’s age, mean (SD)	11.56 (2.90)	11.48 (2.98)	11.64 (2.82)	0.477
Mother/informant: age, mean (SD)	37.07 (8.24)	37.08 (8.79)	37.06 (7.67)	0.977
Number of people residing in the household, mean (SD)	4.24 (1.18)	4.24 (1.21)	4.24 (1.15)	1.000
Number of children in the household aged 6–15 years, mean (SD)	1.44 (0.66)	1.42 (0.70)	1.45 (0.62)	0.64
Family purchase power score, mean (SD)	13.28 (4.75)	13.04 (4.94)	13.52 (4.55)	0.192
Household receive water from the public system, n (%)				0.466
No	502 (76.1)	255 (77.3)	247 (74.8)	
Yes	158 (23.9)	75 (22.7)	83 (25.2)	
Mother: worked for pay in last 30 days, n (%)				1.000
No	296 (44.9)	148 (44.8)	148 (44.8)	
Yes	364 (55.2)	182 (55.2)	182 (55.2)	
Mother living with a husband/partner, n (%)				0.874
No	262 (39.7)	130 (39.4)	132 (40.0)	
Yes	398 (60.3)	200 (60.6)	198 (60.0)	

*Note:* BFP, Bolsa Família Program.

* Groups compared using Logistic regression models.

We also examined differences between the 330 BFP beneficiaries in the matched sample and the 43 excluded beneficiaries from the original group. Appendix 3.5 (Table 3.5) suggests the matching process tended to exclude participants with a greater likelihood of participation and more socioeconomic disadvantage (lower purchasing power, larger household size, and higher maternal unemployment), due to the challenge of finding comparable non-participants with similar characteristics.

Sociodemographic characteristics of the matched sample, distinguishing between participants and non-participants of the BFP, are presented in Table 4.

### Association between BFP, adversity exposure, and emotional and behavioral problems in the matched sample

Table 5 presents clinical characteristics of the matched sample. Linear regression models indicated that higher adversity scores at baseline were associated with higher total (β = 0.39, SE = 0.98, p < 0.001), internalizing (β = 0.39, SE = 0.93, <0.001), and externalizing (β = 0.35, SE = 0.96, p < 0.001) CBCL T-scores at baseline. BFP beneficiaries reported higher exposure to adversity β = 0.10, SE = 0.03, p = 0.010) compared to families who did not receive BFP. The frequency of exposure to each adverse event by BFP status is detailed in Appendix 4. BFP participants reported greater exposure to seeing a dead person as a victim of violence (5.8% versus 1.8%), and problems with alcohol or drugs in a close family member (17.9% versus 10%) compared with non-beneficiaries.

**Table 5.** Clinical characteristics of the sample: Child Behavior Checklist T-scores and standardized factor scores of adversity index by BFP participation (n = 660)

Child Behavior Checklist scores	Total (n = 660)		Non-BFP (n = 330)		BFP (n = 330)	
	Baseline	Follow-up	Baseline	Follow-up	Baseline	Follow-up
CBCL total score, mean (SD)	50.93 (11.56)	50.35 (10.37)	49.66 (11.14)	48.40 (10.00)	52.20 (11.85)	52.31 (10.38)
CBCL internalizing score, mean (SD)	52.33 (11.01)	52.49 (9.44)	51.14 (10.69)	50.78 (9.40)	53.52 (11.20)	54.21 (9.19)
CBCL externalizing score, mean (SD)	51.65 (11.21)	50.63 (10.28)	50.46 (10.59)	49.00 (9.77)	52.85 (11.69)	52.28 (10.53)
Standardized adversity factor scores, mean (SD)	−0.06 (0.42)	-	−0.11 (0.41)	-	−0.02 (0.43)	-

*Note*: BFP, Bolsa Família Program; CBCL, Child Behavior Checklist

### Moderation effect of BFP on the association between adversity exposure and changes in CBCL T-scores

**Figure 1.**
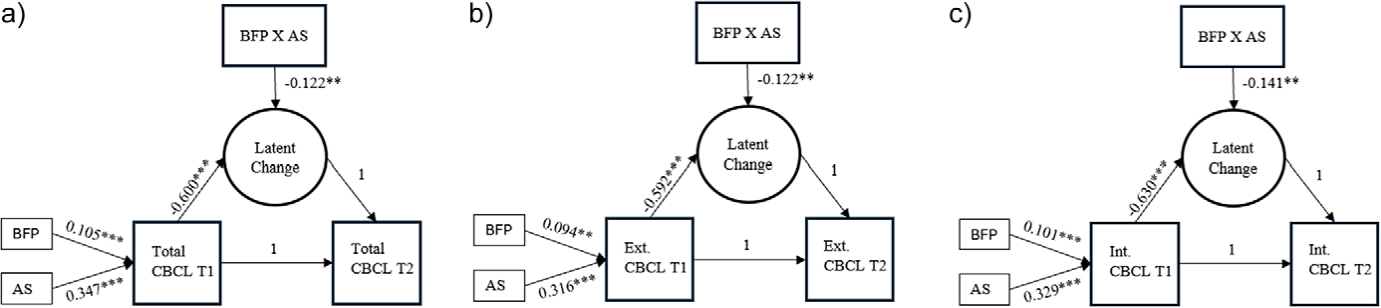
Latent change score (LCS) model for changes in total (a), externalizing (b) and internalizing (c) CBCL T-Scores between baseline and follow-up. Standardized coefficients are presented, **p < 0.05, ***p < 0.001. *Note*: AS, adversity scores; BFP, Bolsa Família Program; BFP X AS, interaction between *Bolsa Família* Program and adversity scores; CBCL, Child Behavior Checklist; T1, baseline; T2, follow-up. Fit index results: (a) RMSEA = 0.050, CFI = 0.976, TLI = 0.944; b) RMSEA = 0.049, CFI = 0.976, TLI = 0.944; c) RMSEA = 0.066, CFI = 0.959, TLI = 0.904.

Table 6 presents results of LCSMs. We observed a reduction in total (mean slope = −0.536, SE = 0.458) and externalizing (mean slope = −0.989, SE = 0.449) CBCL T-scores, and an increase in internalizing CBCL T-scores (mean slope = 0.198, SE = 0.433) between the two time points. As shown in Table 6, no association was observed between BFP and changes in total, externalizing, and internalizing CBCL T-scores between baseline and follow-up. In Figure 1, it is observed that both BFP participation and adversity scores were significantly associated with higher CBCL T-scores at baseline, indicating that children from more adverse backgrounds and those receiving BFP started with elevated levels of emotional and behavioral symptoms. Across all models, the proportional change coefficients were negative, suggesting that participants with higher baseline symptom levels experienced smaller reductions in symptoms over time. A significant interaction between adversity and BFP participation was observed, indicating that BFP participation moderated the relationship between adversity and changes in symptoms. Specifically, the interaction was associated with reductions in total problems (β = −0.124, SE = 0.034, p < 0.001), externalizing problems (β = −0.122, SE = 0.036, p = 0.001), and internalizing problems (β = −0.141, SE = 0.033, p < 0.001). These findings suggest that for each one standard deviation increase in exposure to adversity, participation in the program was associated with a reduction of 0.12 standard deviations in total and externalizing mental health problems, and a 0.14 standard deviation reduction in internalizing problems at follow-up.

**Table 6.** Predictors of latent change scores from baseline and follow-up in the matched sample, standardized β coefficients (n = 660)

Predictor	Latent change score total CBCL-T scores			Latent change score ExternalizingCBCL-T scores			Latent change score Internalizing CBCL-T scores		
	β	SE	p value	Β	SE	p value	β	SE	p value
Bolsa Família participation at baseline	0.057	0.039	0.140	0.039	0.039	0.313	0.046	0.039	0.234
Bolsa Família × adversity scores interaction	−0.124	0.034	<0.001	−0.122	0.036	0.001	−0.141	0.033	<0.001

*Note*: CBCL, Child Behavior Checklist; SE, standard error.

### Sensitivity analyses

Table 7 presents the results of sensitivity analyses that corroborated that the interaction between BFP and adversity was associated with lower level of mental health problems at follow-up in the following conditions:

1. Operationalizing BFP participation duration as a continuous variable, indicating that longer participation in the program, combined with higher adversity exposure, was associated with lower total, externalizing, and internalizing CBCL T-scores at follow-up.
2. Operationalizing BFP participation using a lower threshold of 6 months, suggested that even including participants that received the benefit for a more limited duration (n = 22), we identified an association with lower total, externalizing, and internalizing CBCL T-scores at follow-up among participants exposed to higher levels of adversity.
3. Using the full sample before matching, the results remain significant but, as shown in Table 7, a smaller difference in mental health problems was observed.
4. Excluding the ABEP scores and piped water access from the matching.
5. When excluding IPWs intended to handle attrition.

**Table 7.** Sensitivity analyses of interaction between participation in Bolsa Família Program and adversity factor score

Predictor	Latent change score Total CBCL-T scores			Latent change score Externalizing CBCL-T scores			Latent change score Internalizing CBCL-T scores		
	Β	SE	p value	Β	SE	p value	β	SE	p value
Months of participation in Bolsa Família × adversity scores interaction (n = 660)	−0.131	0.042	0.002	−0.130	.043	0.003	−0.131	0.038	<0.001
Bolsa Família for at least 6 months × adversity scores interaction (n = 660)	−0.113	0.041	0.006	−0.114	0.041	0.005	−0.133	0.035	<0.001
Bolsa Família × adversity scores interaction. Unmatched sample (n = 1120)	−0.073	0.025	0.004	−0.069	0.027	0.011	−0.093	0.024	<0.001
Bolsa Família × adversity scores interaction. Matching excluding ABEP scores (n = 706)	−0.107	0.032	−0.001	−0.119	0.034	0.001	−0.109	0.031	<0.001
Bolsa Família × adversity scores interaction. No adjustment for missing data bias (n = 660)	−0.124	0.034	<0.001	−0.122	0.036	0.001	−0.142	0.033	<0.001

*Note*: CBCL-T, Child Behavior Checklist T-scores; SE, standard error.

## Discussion

This study assessed the association between participation in the BFP and the mental health of children and adolescents, with a particular focus on those exposed to higher levels of adversity. Our findings indicate that while no significant association was found in the overall sample, notable associations emerged in the subgroup exposed to greater adversity. Specifically, children and adolescents facing higher levels of violence and other social adversities showed smaller declines in total, internalizing, and externalizing mental health problems over time. This suggests that the BFP may help mitigate the negative effects of accumulated adversities, with those in particularly challenging contexts – beyond the poverty experienced by all BFP beneficiaries – possibly deriving greater benefits from the program. Individuals with lower socioeconomic status are also more likely to experience higher levels of trauma and violence [7, 30, 60], and this subgroup may benefit more from the income relief provided by the BFP. Therefore, the modest positive associations observed in our study may be more pronounced among this more vulnerable subgroup.

Previous research analyzing the impact of CTP on child and adolescent mental health is mixed. Most studies show small positive effects, particularly on internalizing symptoms [31, 32], while others detect no effect [61, 62]. A potential explanation is that CTPs confer modest overall benefits, which may be difficult to detect in a broad study population. However, these benefits appear more pronounced in vulnerable subgroups. In our study, even though the association was small, it was positive across all outcomes among these vulnerable groups.

Several pathways have been proposed to explain how CTPs might benefit child and adolescent mental health [6, 8, 63]. First, provision of regular financial support may reduce household economic uncertainty mitigating stress experienced by children and families [26, 27, 63–65]. Second, consistent income may help families meet basic needs which can diminish the stigma and shame associated with poverty [66–68]. Third, mainly qualitative evidence from a review of CTPs in Africa suggests that these programs strengthen social capital, such as trust and community engagement [18], thereby reducing additional stressors linked to violence and social marginalization. Fourth, the BFP may enhance household functioning, as demonstrated by a study of South Africa’s Child Support Grant, which benefits over 60% of the country’s children. The study showed that participation contributed to mothers’ engagement in children’s activities, positively impacting child well-being [69]. An indirect benefit of BFP in this scenario would be the reduction of adverse childhood experiences, especially among those facing multiple adversities, thereby enhancing household functioning [70, 71]. Together, these mechanisms may be crucial for families facing multiple adversities, like the most vulnerable participants in our sample. Reliable financial resources can buffer against the cumulative negative effects of socioeconomic and environmental challenges [6, 8, 63]. All these potential explanations are important for understanding the complexity of how cash transfer programs may positively influence the mental health of children and adolescents. However, we acknowledge that our observational study design does not allow us to identify or confirm the specific mechanisms involved. These pathways should therefore be interpreted as hypotheses rather than direct findings from our data. Future research, particularly longitudinal or mixed-methods studies, is needed to investigate these mechanisms in greater depth.

Our findings also indicate a small but significant association between BFP participation and fewer internalizing problems among children and adolescents who experienced heightened exposure to violence and other social adversities. These results support the growing body of evidence that CTPs may alleviate depressive/internalizing symptoms among young people [31, 33] and, importantly, highlight this benefit is most pronounced for those exposed to greater adversity.

Additionally, the study found a positive association between BFP participation and lower levels of externalizing problems among the most vulnerable children/adolescents – a noteworthy contribution given the scarcity of data assessing this outcome. The systematic review mentioned previously [31] found only three randomized controlled trials that assessed externalizing problems or behavioral functioning. All were conducted in Latin America over a decade ago [72–74] and faced limitations by using non-validated instruments to assess externalizing problems [73] or being focused on specific rural populations [72, 74]. Among these, two showed a small reduction in externalizing problems, while one found no discernible effect [74]. Subsequent research on BFP, using quasi-experimental techniques and propensity score matching, found no impact on externalizing problems [62]. Our study contributes to this limited body of research by suggesting a potential association between participation in the BFP and lower levels of externalizing problems among highly vulnerable youth.

### Strengths and limitations

Our study provides novel insights into the role of CTPs in the mental health of children/adolescents by considering a broad spectrum of mental health outcomes, analyzing different levels of accumulated adversities within a matched community sample, and employing standardized and validated instruments to measure mental health problems.

However, primarily related to the observational design of the study, including the use of only two waves spaced 12 months apart, which limits the ability to assess the long-term stability of the findings. For example, a recent systematic review indicates positive results of CTP in reducing depression and anxiety among adults in developing countries but suggests this benefit is not sustained in the medium to long term without the continued provision of cash [29].

The sample was drawn from a single Brazilian municipality, which limits its representativeness and may affect the generalizability of the findings. Additionally, we found a consistent, albeit small, negative association between BFP participation and total, internalizing, and externalizing mental health problems among subgroups more exposed to violence and other social adversities. However, we acknowledge that the stratified analyses led to smaller subgroup sizes, potentially compromising statistical power and contributing to the modest associations observed.

This study cannot address the mechanisms between the BFP and identified mental health problems. Hence, our discussion is based on hypotheses from previous studies without empirical confirmation. Future research should investigate whether the positive effects arise directly from poverty alleviation, indirectly through impacts on parents, or distally through community improvements. Several studies suggest that effects on mental and physical health vary due to individual temperament, with some being more sensitive to their environment. Factors such as the type, amount, duration, and timing of adversity also influence outcomes. Often, it is a combination of factors, as few people experience only one form of adversity at a time [71].

In conclusion, while the BFP has been a well-established initiative for over 20 years, presently benefiting 21 million families, it was not originally designed with the objective of enhancing mental health outcomes. This may explain why some studies have not identified positive effects on the mental health of children/adolescents [61, 62], while our study observed only a modest effect limited to the most vulnerable populations. By capturing its positive impact on this specific group, our data support its continuation and prioritization for the most vulnerable beneficiaries, virtually without additional costs.

The main contribution of this study lies in identifying an association between participation in the CTP and mental health outcomes among children and adolescents exposed to higher levels of accumulated adversity. While the overall association was small and not observed across the entire sample, the moderation effect found among more vulnerable subgroups suggests that targeted social protection policies may support youth mental health in contexts of chronic poverty. This study was conducted in one city in Brazil, and further research should explore how this might generalize more widely, including in other LMICs with established conditional cash transfer programs.

## Supporting information

10.1192/j.eurpsy.2025.10109.sm001 Paula et al. supplementary material Paula et al. supplementary material

## Data Availability

Data were provided by the Brazilian High-Risk Cohort study and are available upon request in the Open Science Framework public repository (https://osf.io/ktz5h/).

## Supplementary material

The supplementary material for this article can be found at http://doi.org/10.1192/j.eurpsy.2025.10109.
