# Supplementary material for: Can cash transfers protect mental health? Evidence from an observational cohort of children and adolescents living in adverse contexts in Brazil
Source: Eur Psychiatry. 2025 Sep 24;68(1):e145. doi: 10.1192/j.eurpsy.2025.10109 (PMC12538174; doi:10.1192/j.eurpsy.2025.10109)
Supplement: Paula et al. supplementary material [file S0924933825101090sup001.zip › Final Appendix 1_Figure CFA Diagram_Aug14.pdf]

Appendix 1

Summary

Diagram 1. Latent Change Score Model Diagram: Predicting change in Child Behavior Checklist (CBCL) scores from the interaction between adversity factor scores and *Bolsa Família* Program participation **Page 2**

Diagram 1 Confirmatory Factor Analysis: One-factor structure of the adversity assessment and standardized factor loadings (Itaboraí Youth Study, baseline, n=1409)

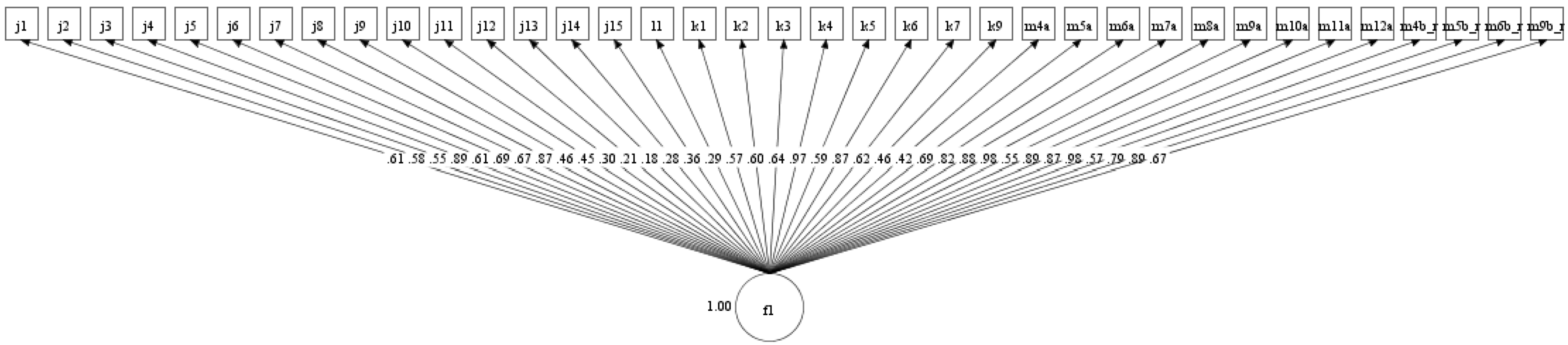

Model fit Index: RMSEA 0.035, CFI =0.932, TLI =0.928 (Acceptable model fit indexes are: RMSEA<0.08, CFI and TLI>0.90).
