# Supplementary material for: Can cash transfers protect mental health? Evidence from an observational cohort of children and adolescents living in adverse contexts in Brazil
Source: Eur Psychiatry. 2025 Sep 24;68(1):e145. doi: 10.1192/j.eurpsy.2025.10109 (PMC12538174; doi:10.1192/j.eurpsy.2025.10109)
Supplement: Paula et al. supplementary material [file S0924933825101090sup001.zip › Final Appendix 2_Figure Latent Change_Aug14.pdf]

Appendix 2

Summary

Figure 2. Latent Change Score Model Diagram: Predicting change in Child Behavior Checklist (CBCL) scores from the interaction between adversity factor scores and *Bolsa Família* Program participation **Page 2**

Figure 2 Latent Change Score Model Diagram: Predicting change in Child Behavior Checklist (CBCL) scores from the interaction between adversity factor scores and *Bolsa Família* Program participation

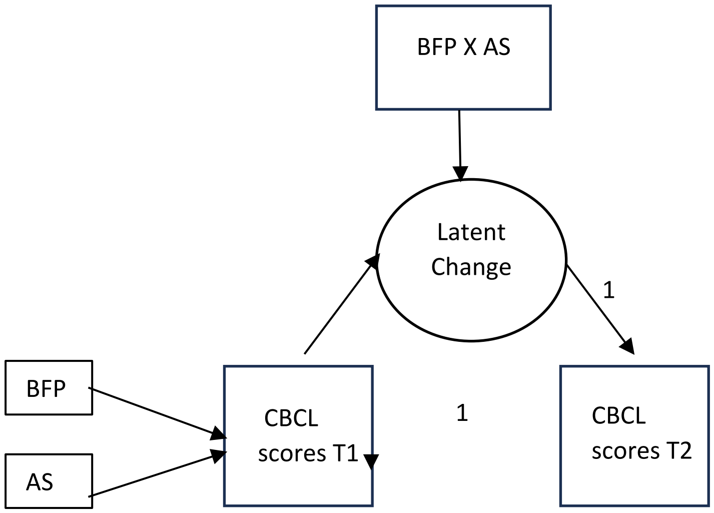

- Observed variables, Bolsa Família Program (BFP) participation, adversity scores (AS) and outcome scores at T1 and T2, are shown as rectangles.
- A latent change factor is shown as a circle and represents the unobserved change from T1 to T2.
- A path from T1 to T2 is fixed at 1, indicating that T2 is modeled as a function of T1 plus the latent change factor.
- The latent change factor loads onto the outcome at T2 with a fixed value of 1, meaning it directly contributes to T2 scores.
- A covariance path is included between the T1 score and the latent change factor, allowing for the possibility of proportional change (i.e., that change depends on baseline level).
- Exogenous predictors (e.g., Bolsa Família Program participation, adversity index) are shown as variables with arrows pointing to T1 (predicting initial level).
- An interaction term between adversity and BFP participation is included as a predictor of the latent change factor to test moderation effects.
