# Supplementary material for: Can cash transfers protect mental health? Evidence from an observational cohort of children and adolescents living in adverse contexts in Brazil
Source: Eur Psychiatry. 2025 Sep 24;68(1):e145. doi: 10.1192/j.eurpsy.2025.10109 (PMC12538174; doi:10.1192/j.eurpsy.2025.10109)

Figure 3.3. Family purchase power score by Bolsa Família participation: Density plot before matching

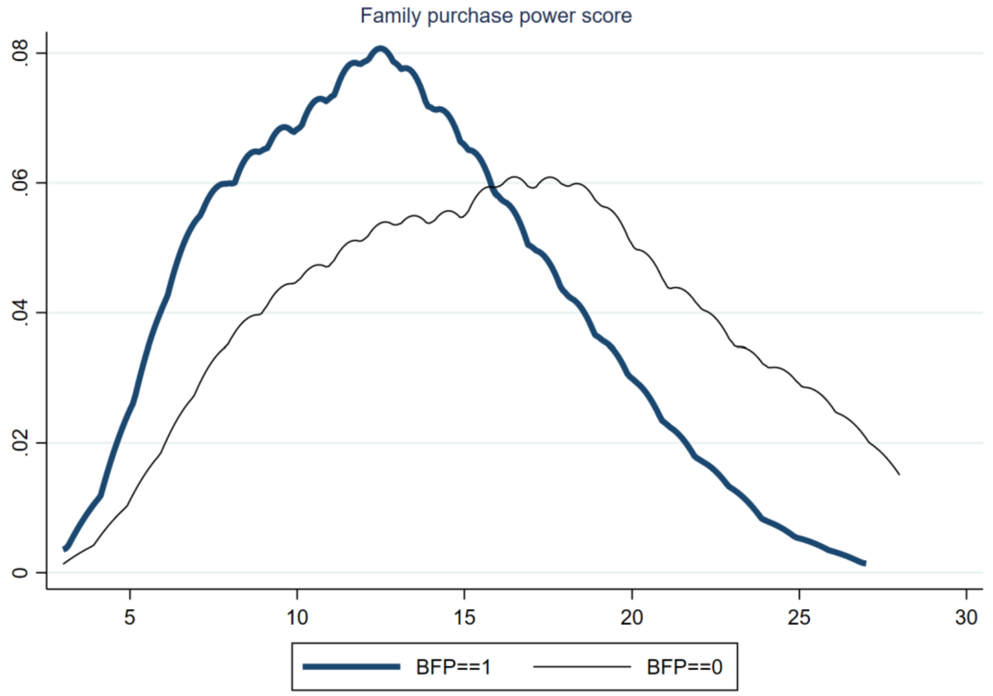

Figure 3.4. Family purchase power scores by Bolsa Família participation: Density plot after matching

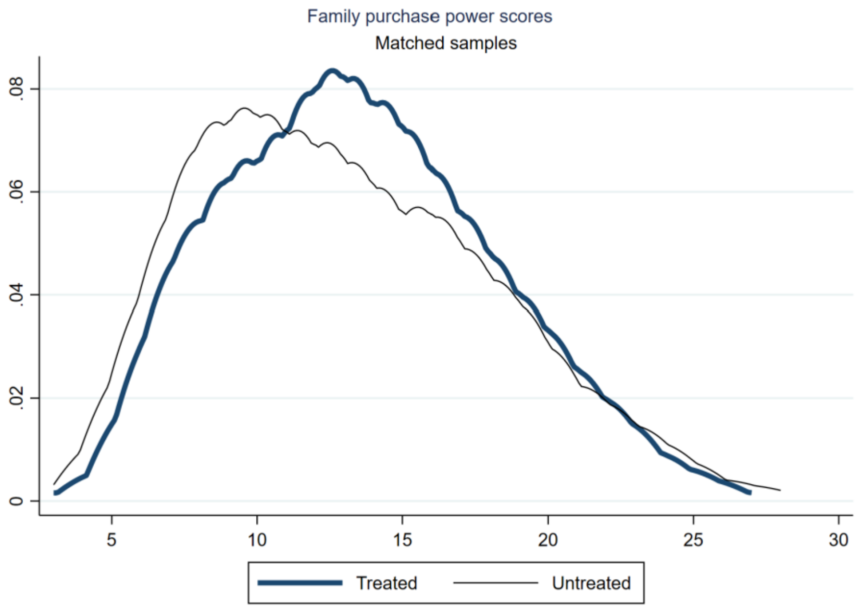

Supplement: Paula et al. supplementary material [file S0924933825101090sup001.zip › Final Appendix 3.3. 3.4. Figures Family purchase.pdf]
