# Supplementary material for: Can cash transfers protect mental health? Evidence from an observational cohort of children and adolescents living in adverse contexts in Brazil
Source: Eur Psychiatry. 2025 Sep 24;68(1):e145. doi: 10.1192/j.eurpsy.2025.10109 (PMC12538174; doi:10.1192/j.eurpsy.2025.10109)
Supplement: Paula et al. supplementary material [file S0924933825101090sup001.zip › FINAL Appendix 4. Table adverse event.docx]

**Appendix 4**

Summary Page

Table 4 Frequency of exposure to each adversity at baseline by BFP participation status 2

**Table 4 Frequency of exposure to each adversity at baseline by BFP participation status (n=660)**

| **Item** | **Total** | **Non-BFP** | **BFP** | **p value*** |
| --- | --- | --- | --- | --- |
| Building collapse | 9 (1.4%) | 5 (1.5%) | 4 (1.2%) | 0.74 |
| Fire, flood or other natural disaster | 3 (0.5%) | 2 (0.6%) | 1 (0.3%) | 0.56 |
| Life-threatening accident | 10 (1.5%) | 6 (1.8%) | 4 (1.2%) | 0.52 |
| Running over with serious injury | 1 (0.2%) | 0 (0.0%) | 1 (0.3%) | 0.32 |
| Biking accident with serious injury | 7 (1.1%) | 4 (1.2%) | 3 (0.9%) | 0.70 |
| Motorcycle accident with serious injury | 2 (0.3%) | 1 (0.3%) | 1 (0.3%) | 1.00 |
| Car accident with serious injury | 2 (0.3%) | 0 (0.0%) | 2 (0.6%) | 0.16 |
| Other traffic accident with serious injury | 1 (0.2%) | 0 (0.0%) | 1 (0.3%) | 0.32 |
| Child saw a dead person victim of violence | 23 (3.5%) | **6 (1.8%)** | **17 (5.2%)** | **0.020** |
| Child saw a dead person victim of accident | 23 (3.5%) | 8 (2.4%) | 15 (4.5%) | 0.14 |
| Child received bad news about violent death or serious injury of a loved on | 65 (9.8%) | 34 (10.3%) | 31 (9.4%) | 0.70 |
| Life-threatening illness of a close family member | 107 (16.2%) | 46 (13.9%) | 61 (18.5%) | 0.11 |
| Death of a close family member | 126 (19.1%) | 56 (17.0%) | 70 (21.2%) | 0.17 |
| Problems with alcohol or drugs of a close family member | 92 (13.9%) | **33 (10.0%)** | **59 (17.9%)** | **0.003** |
| Close family member being arrested or having problems with the police | 58 (8.8%) | 26 (7.9%) | 32 (9.7%) | 0.41 |
| Being attacked or stabbed with a knife | 0 (0.0%) | 0 (0.0%) | 0 (0.0%) | - |
| Being shot | 0 (0.0%) | 0 (0.0%) | 0 (0.0%) | - |
| Being sexually molested by someone much older | 0 (0.0%) | 0 (0.0%) | 0 (0.0%) | - |
| Threatened, maltreated or chased by peers at school | 58 (8.8%) | 22 (6.7%) | 36 (10.9%) | 0.054 |
| Being mugged | 10 (1.5%) | 5 (1.5%) | 5 (1.5%) | 1.00 |
| Someone has broken into or tried to force their way into the house or apartment | 6 (0.9%) | 1 (0.3%) | 5 (1.5%) | 0.10 |
| Being chased by gangs or individuals | 4 (0.6%) | 2 (0.6%) | 2 (0.6%) | 1.00 |
| Being picked-up/arrested by the police or taken to the police station | 0 (0.0%) | 0 (0.0%) | 0 (0.0%) | - |
| Being threatened by someone with serious physical harm | 6 (0.9%) | 1 (0.3%) | 5 (1.5%) | 0.10 |
| Suffering death threats | 1 (0.2%) | 0 (0.0%) | 1 (0.3%) | 0.32 |
| Being beaten-up (outside home/school) | 4 (0.6%) | 1 (0.3%) | 3 (0.9%) | 0.32 |
| Being around a shoot-out | 22 (3.3%) | 9 (2.7%) | 13 (3.9%) | 0.39 |
| Mother hit him/her with an object such as a stick, bloom, cane or belt | 51 (7.7%) | 24 (7.3%) | 27 (8.2%) | 0.66 |
| Mother kicked him/her | 3 (0.5%) | 2 (0.6%) | 1 (0.3%) | 0.56 |
| Mother choked him/her by putting hands (or something else) around his/her neck | 2 (0.3%) | 2 (0.6%) | 0 (0.0%) | 0.16 |
| Mother smothered him/her with hand of pillow | 2 (0.3%) | 2 (0.6%) | 0 (0.0%) | 0.16 |
| Mother burned, scalded or branded him/her | 1 (0.2%) | 1 (0.3%) | 0 (0.0%) | 0.32 |
| Mother beat him/her | 21 (3.2%) | 7 (2.1%) | 14 (4.2%) | 0.12 |
| Mother beat him/her (hit over and over again with object or fist) | 2 (0.3%) | 2 (0.6%) | 0 (0.0%) | 0.16 |
| Mother threatened him/her with a knife or gun | 1 (0.2%) | 1 (0.3%) | 0 (0.0%) | 0.32 |
| Mother harmed him/her with a knife or gun | 1 (0.2%) | 1 (0.3%) | 0 (0.0%) | 0.32 |
| Father hit him/her with an object such as a stick, bloom, cane or belt | 10 (1.5%) | **2 (0.6%)** | **8 (2.4%)** | 0.056 |
| Father kicked him/her | 3 (0.5%) | 1 (0.3%) | 2 (0.6%) | 0.56 |
| Father choked him/her by putting hands (or something else) around his/her neck | 1 (0.2%) | 1 (0.3%) | 0 (0.0%) | 0.32 |
| Father beat him/her | 5 (0.8%) | 3 (0.9%) | 2 (0.6%) | 0.65 |
| Father smothered him/her with hand of pillow | 0 (0.0%) | 0 (0.0%) | 0 (0.0%) | - |
| Father burned, scalded or branded him/her | 0 (0.0%) | 0 (0.0%) | 0 (0.0%) | - |
| Father beat him/her (hit over and over again with object or fist) | 0 (0.0%) | 0 (0.0%) | 0 (0.0%) | - |
| Father threatened him/her with a knife or gun | 0 (0.0%) | 0 (0.0%) | 0 (0.0%) | - |
| Father harmed him/her with a knife or gun | 0 (0.0%) | 0 (0.0%) | 0 (0.0%) | - |

* Groups compared using Pearson's chi-squared
