# Supplementary material for: Can cash transfers protect mental health? Evidence from an observational cohort of children and adolescents living in adverse contexts in Brazil
Source: Eur Psychiatry. 2025 Sep 24;68(1):e145. doi: 10.1192/j.eurpsy.2025.10109 (PMC12538174; doi:10.1192/j.eurpsy.2025.10109)
Supplement: Paula et al. supplementary material [file S0924933825101090sup001.zip › Final Appendix_3.1_Table 3.1 Bias diagnosis.docx]

**Table 3.1. Bias diagnosis before and after matching.**

| Unmatched | Mean %bias | t- test | V(T)/ |  |  |
| --- | --- | --- | --- | --- | --- |
| Variable Matched | Treated Control %bias reduction | t p> t | V (C) |  |  |
| Mother’s age U | 36.71 38.05 -16.4 | -2.53 0.012 | 0.74* |  |  |
| M | 37.06 37.08 -0.2 98.6 | -0.03 0.977 | 0.76* |  |  |
| Number residents U | 4.38 3.97 34.5 | 5.58 0.000 | 1.35* |  |  |
| M | 4.24 4.24 0.0 100.0 | -0.00 1.000 | 0.90 |  |  |
| Purchase power U | 12.96 16.33 -64.3 | -9.80 0.000 | 0.66* |  |  |
| M | 13.52 13.04 9.2 85.7 | 1.30 0.193 | 0.85 |  |  |
| Piped water U | 0.24 0.30 -12.9 | -2.01 0.045 | . |  |  |
| M | 0.25 0.23 5.5 57.6 | 0.73 0.466 | . |  |  |
| Mother worked U | 0.50 0.64 -28.0 | -4.44 0.000 | . |  |  |
| M | 0.55 0.55 0.0 100.0 | 0.00 1.000 | . |  |  |
| Number of children U | 1.54 1.31 34.7 | 5.61 0.000 | 1.36* |  |  |
| M | 1.45 1.42 3.6 89.5 | 0.47 0.638 | 0.79* |  |  |
| * if variance ratio outside [0.82; 1.23] for U and [0.81; 1.24] for M   \| Sample \| Ps R2 \| LR chi2 \| p>chi2 \| MeanBias \| MedBias \| B \| R \| %Var \| \| --- \| --- \| --- \| --- \| --- \| --- \| --- \| --- \| --- \| \| Unmatched \| 0.101 \| 144.59 \| 0.000 \| 31.8 \| 31.2 \| 79.4* \| 0.91 \| 100 \| \| Matched \| 0.002 \| 1.97 \| 0.923 \| 3.1 \| 1.9 \| 10.9 \| 0.89 \| 50 \|   * if B>25%, R outside [0.5; 2]  U=Unmatched, M=Marched | | | | | |
