# Supplementary material for: Can cash transfers protect mental health? Evidence from an observational cohort of children and adolescents living in adverse contexts in Brazil
Source: Eur Psychiatry. 2025 Sep 24;68(1):e145. doi: 10.1192/j.eurpsy.2025.10109 (PMC12538174; doi:10.1192/j.eurpsy.2025.10109)
Supplement: Paula et al. supplementary material [file S0924933825101090sup001.zip › Final Appendix_3.5_Table 3.5 Differences participants.docx]

**Table 3.5. Differences between BFP participants included and excluded in the matching.**

|  | **BFP not matched** | **BFP matched** |  |
| --- | --- | --- | --- |
| **Factor n (%)** | 43 (11.5%) | 330 (88.5%) |  |
| **Child's gender** |  |  | 0.23 |
| **Male** | 19 (44.2%) | 178 (53.9%) |  |
| **Female** | 24 (55.8%) | 152 (46.1%) |  |
| **Mother/informant: age, mean (SD)** | 34.00 (5.47) | 37.06 (7.67) | 0.012 |
| **Number of people residing in the household, mean (SD)** | 5.51 (1.62) | 4.24 (1.15) | <0.001 |
| **Family purchase power score, mean (SD)** | 8.65 (3.21) | 13.52 (4.55) | <0.001 |
| **Household receive water from the public system** |  |  | 0.35 |
| **No** | 35 (81.4%) | 247 (74.8%) |  |
| **Yes** | 8 (18.6%) | 83 (25.2%) |  |
| **Mother: worked for pay in last 30 days** |  |  | <0.001 |
| **No** | 38 (88.4%) | 148 (44.8%) |  |
| **Yes** | 5 (11.6%) | 182 (55.2%) |  |
| **Mother living with a husband/partner** |  |  | 0.35 |
| **No** | 14 (32.6%) | 132 (40.0%) |  |
| **Yes** | 29 (67.4%) | 198 (60.0%) |  |
| **Index child: age, mean (SD)** | 10.81 (2.99) | 11.64 (2.82) | 0.073 |
| **Number of children in the household aged 6-15 years, mean (SD)** | 2.28 (0.93) | 1.45 (0.62) | <0.001 |
