# Supplementary figures and images for: Can cash transfers protect mental health? Evidence from an observational cohort of children and adolescents living in adverse contexts in Brazil
Source: Eur Psychiatry. 2025 Sep 24;68(1):e145. doi: 10.1192/j.eurpsy.2025.10109 (PMC12538174; doi:10.1192/j.eurpsy.2025.10109)

**Figure 3.1. Standardized percentage of bias across covariates before and after matching.**

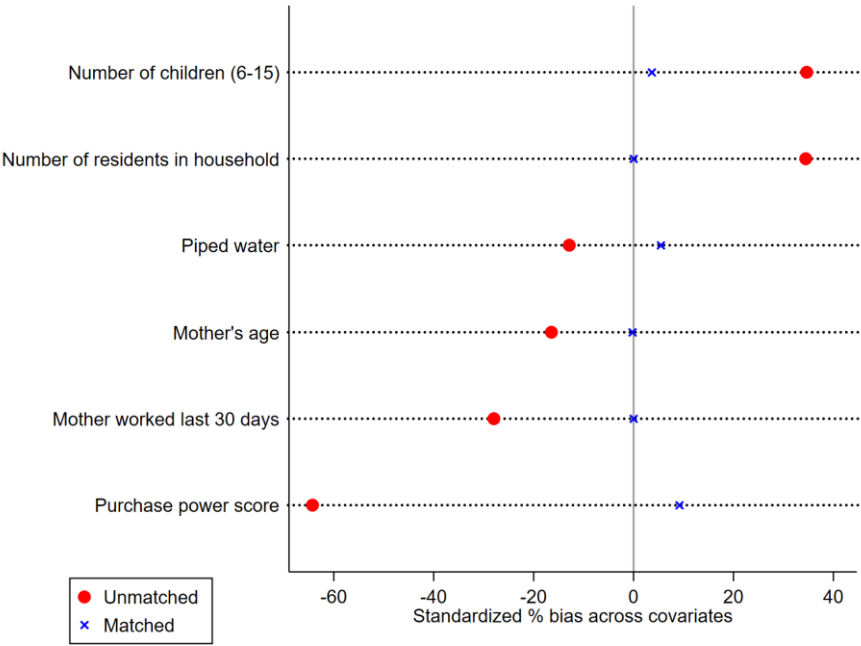

Supplement: Paula et al. supplementary material [file S0924933825101090sup001.zip › Final Appendix 3.1_Figure 3.1 Standardized percentage.pdf]

Figure 3.2 Density plot of BFP participation propensity score before and after matching

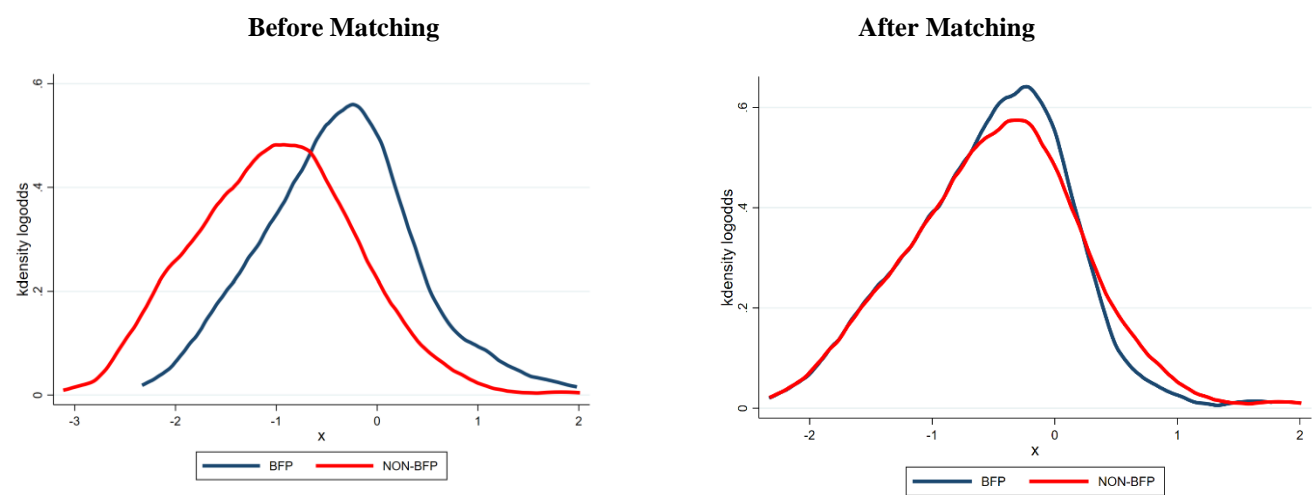

Supplement: Paula et al. supplementary material [file S0924933825101090sup001.zip › Final Appendix 3.2_Figure 3.2. Density plot.pdf]
